# CT Diagnosis of a Thoracic Aort Aneurysm with Type B Aortic Dissection Clinically Misdiagnosed as Acute Pulmonary Embolism

**DOI:** 10.1155/2012/720394

**Published:** 2012-09-09

**Authors:** Ahmet Mesrur Halefoglu

**Affiliations:** Department of Radiology, Sisli Etfal Training and Research Hospital, Sisli, 34360 Istanbul, Turkey

## Abstract

A 54-year-old man was admitted to the emergency department, presenting with an acute onset of chest pain and severe respiratory distress symptoms. He was medicated with intravenous analgesia and antihypertensive drugs. The patient was subjected to a chest X-ray which revealed a prominent widening of the mediastinum and pleural effusion on the left side. In laboratory tests-d-dimer level was highly elevated. The patient was clinically interpreted as having an acute pulmonary embolism and referred to the radiology clinic to perform a computed tomography (CT) examination. Contrast-enhanced CT demonstrated that there was no abnormality related to the pulmonary vasculature, but a huge thoracic aorta aneurysm measuring 11 × 8.1 × 7.7 cm in diameter was detected. Accompanying the aneurysm, an intimal flap was also present in the proximal descending thoracic aorta, distal to the origin of the left subclavian artery and extending into the bifurcation level. The patient was therefore diagnosed as having a type B aortic dissection as well. Once these serious conditions were detected, he was immediately transferred to a cardiovascular thoracic surgery hospital for endovascular repairment operation.

## 1. Introduction

Chest pain is a common and challenging clinical problem. Three important life-threatening causes of chest pain are aortic dissection (AD), pulmonary embolism (PE), and acute coronary syndrome [1]. Unstable thoracic aneurysms and pseudoaneurysms can also produce chest pain that may radiate to the jaw or back. AD is the most common acute emergency condition of the aorta, often leading to death. The incidence of AD has been reported to be 2,000 new cases per year in the United States and 3,000 in Europe [2, 3]. It has an estimated 1%-2% per hour mortality rate within the first 24 hours after onset and an 80% rate of untreated mortality at two weeks [4]. 

CT is an effective tool for the assessment of chest pain in the acute setting. It is fast, available, and safe. Diagnostic accuracy for the detection of acute aortic dissection (AAD) and PE is superior to other competing methods [5].

## 2. Case Report

A 54-year-old man complaining of abrupt chest pain and respiratory distress was admitted to our emergency department. His physical examination revealed a blood pressure of 260/140 mmHg, respiratory rate of 28 breaths/min and a pulse of 106 beats/min The patient had a history of hypertension. He was medicated with intravenous analgesia and antihypertensive drugs by the internal medicine department. His pain had an abrupt tearing fashion on the center of his chest and had been radiating to the posterior thoracic region for the last couple of hours. The patient's electrocardiography (ECG) and blood counts were within normal limits, but d-dimer level was elevated (850 ng/mL normal limits: 0–500 ng/mL). A chest X-ray was performed and demonstrated a conspicuous widening of the mediastinum and pleural effusion in the left hemithorax. The patient was clinically diagnosed as having acute PE and referred to CT examination. 

CT examination was performed with a multidetector row helical CT scanner (Somatom Sensation 16, Siemens Medical Systems, Erlangen, Germany). Unenhanced and contrast-enhanced CT images were provided. Contrast-enhanced CT images were obtained following 150 mL of nonionic contrast material administration by using a power injector at a rate of 2–4 mL/sec. Following contrast media administration, CT images were obtained at 20–30 seconds and 120–150 seconds. We covered the area extending from the upper thorax to the iliac arteries level. Axial images with 5 mm section thickness were generated and these images were subjected to reconstruction which yielded 2 mm section thickness multiplanar reformation images (MPR). MPR images included axial, coronal, and sagittal planes. For detailed information we also performed maximum intensity projection (MIP) and 3D volume rendering images.

**Figure 1:**
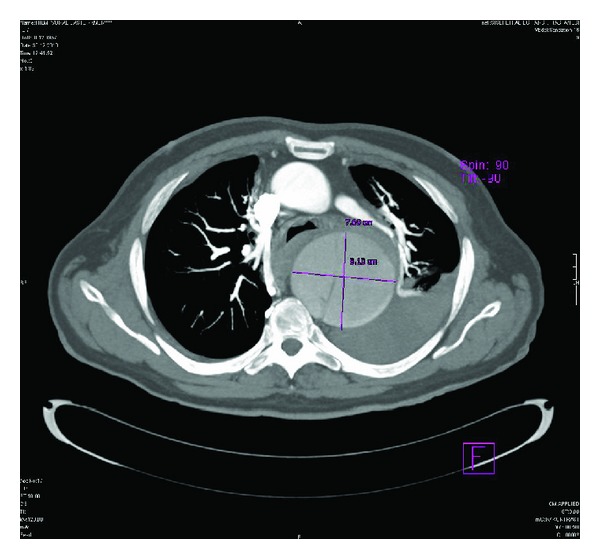
Axial image through thorax, a huge proximal descending thoracic aorta aneurysm with a hypointens intimal flap in it is seen. A perimediastinal hematoma and left pleural effusion are also present.

**Figure 2:**
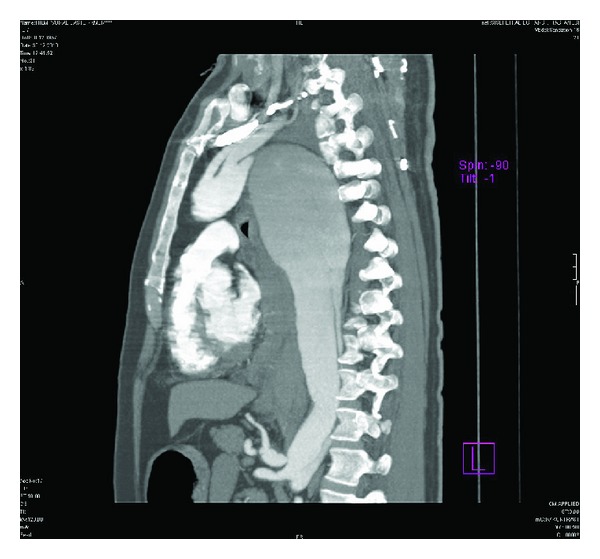
Sagittal MPR image, intimal flap extending from proximal descending thoracic aorta to bifurcation level is demonstrated.

**Figure 3:**
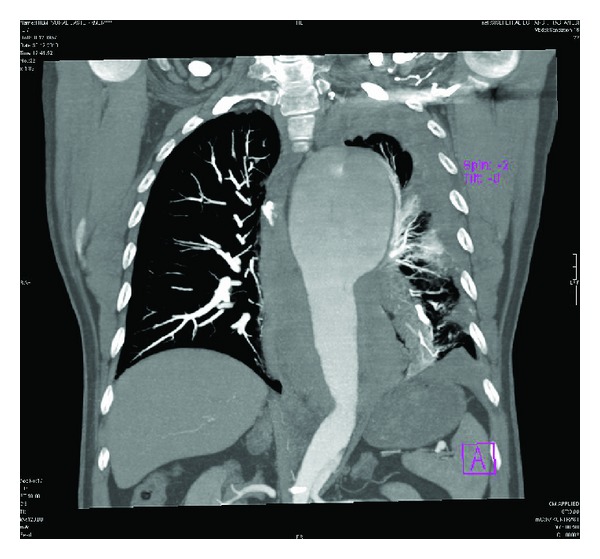
Coronal MPR image, showing thoracic aorta aneurysm and intimal flap.

**Figure 4:**
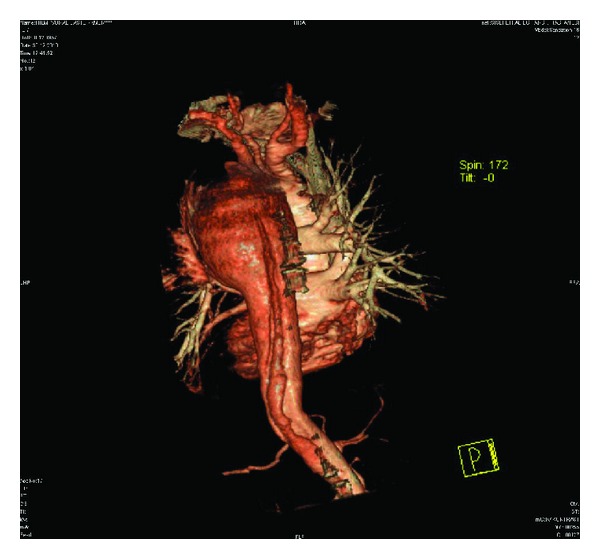
3D volume rendering image, clearly delineating the pathologies.

On these images, a huge aneurysm measuring 11 × 8.1 × 7.7 cm in diameter in the proximal descending thoracic aorta was found. In this aneurysm, a hypointense intimal flap was also detected. Other associated findings were perimediastinal hematoma and hemothorax in the left lung (Figure 1). The intimal flap was extending from the proximal descending thoracic aorta, distal to the left subclavian artery origin to the bifurcation level (Figures 2 and 3). However, both major aortic arch and main abdominal aorta branches were patent and not involved by the intimal flap. The pulmonary vessels were clearly visualized without containing any thrombus material, and this finding led us to rule out an acute PE in the differential diagnosis. Based on these CT findings, a huge proximal descending thoracic aorta aneurysm with type B AD was diagnosed. These pathologies were also clearly demonstrated on 3D volume rendering images (Figure 4).

The patient was immediately transferred to the cardiovascular thoracic surgery department where an endovascular repairment operation was successfully performed.

## 3. Discussion

Aneurysms are diagnosed when the aorta has an abnormal segment with a diameter greater than 50% of the adjacent normal aorta. More commonly, aneurysms are diagnosed when the thoracic aorta exceeds 5 cm in diameter and the abdominal aorta exceeds 3 cm [1].

AD occurs when an intimal tear develops, allowing blood to penetrate the aortic wall, dissects longitudinally through the media, and forms a false lumen [6]. Systemic hypertension is regarded as the major predspsing factor to the development of AD [7]. Another important potential etiology is cystic medial necrosis which can be seen with Marfan's disease. In fact, Marfan's disease accounts for the majority of dissections in patients younger than 40 [7]. Other associations with AD include chromosomal aberrations (Turner's or Noonan's syndrome), bicuspid aortic valve, Ehlers-Danlos syndrome, aortic coarctation, pregnancy, cocaine use, and prior catheterization [8]. Atherosclerosis is not an independent risk factor for AD.

AADs are characterized by symptoms that are present for less than 14 days; in chronic dissections, the symptoms are present for a longer period [9].

The Stanford classification system for dissections is based on the need for surgical intervention. Stanford type A dissection involves the ascending thoracic aorta and the dissection flap may extend into the descending aorta. Type A dissections account for 60%–70% of cases [10] and typically require urgent surgical intervention to prevent extension into the aortic root, pericardium, or coronary arteries [11]. If untreated, type A dissections are associated with a mortality rate of over 50% within 48 hours [12].

Stanford type B dissection involves the descending thoracic aorta distal to the left subclavian artery and accounts for 30%–40% of cases [10]. Management requires medical treatment of hypertension, unless there are complications due to extension of the dissection (e.g., end-organ ischemia or persistent pain) that would necessitate surgical intervention.

Patients typically present with complains of an abrupt onset of chest pain. The location of the pain can migrate as the dissection increases in size. Physical examination findings are limited in many dissection cases. Aortic murmurs can result from proximal dissection. If the dissection migrates proximally into the pericardium, resulting symptoms of tamponade can occur. The dissection can also lead to occlusion of the coronary arteries that can lead to acute myocardial infarctions. These do routinely occur in approximately 5% of Stanford type A dissections [13].

Chest X-rays are useful in the initial evaluation but are not specific for diagnosis. Abnormalities on X-ray occur in 60%–90% of ADs, but if they are not present, the diagnosis cannot be excluded [14]. A widened mediastinum can be present in both Stanford type A and B dissections.

Adding plasma d-dimer measurement to the clinical assessment allows for confident exclusion of AD. Elevated d-dimer, a breakdown product of cross-linked fibrin, is a highly sensitive but nonspecific marker for AD, present in virtually all cases [15]. Electrocardiographic (ECG) findings may be normal unless there has been compromise of the coronary arteries giving rise to an acute coronary syndrome with associated findings on the ECG tracing.

CT is highly sensitive and specific for the detection and characterization of the AD. On unenhanced images, thickening of the aortic wall and displacement of intimal calcifications may be seen. CT findings of the AD center on the detection of intimomedial flap which requires intravenous contrast. When any form of intravascular treatment is planned, distinguishing the true lumen from the false lumen becomes important. In most cases, the true lumen is the one that is continuous with the unondissected portion of the aorta [16]. A larger lumen size on axial imaging is a feature suggestive of the false lumen. Additional CT signs that may be helpful include the beak sign and the aortic cobweb sign. The beak sign refers to an acute angle with the intimomedial flap and the vessel wall and is a feature of the false lumen. The cobweb sign refers to strands of medial tissue that may be seen within the false lumen. When multiple phases of contrast enhancement are obtained, the false lumen tends to enhance late and washes out in a delayed fashion. 

CT is the most frequently used diagnostic imaging modality for the initial evaluation of patients with suspected AD [17]. Multidetector row CT is the most rapid diagnostic test for AD, with data acquisition accomplished in less than 30 seconds [18]. Reported sensitivities and specificities range from 79% to 100% for CT, but early studies should be interpreted with caution as this technology is evolving rapidly [18, 19]. Angiography and echocardiography are often implemented to characterize the lesion in more details. Other imaging modalities can include magnetic resonance imaging (MRI) and ultrasound (US), but they are not as useful in the initial evaluation of aortic dissection. Modern multisection CT allows rapid image acquisition and data reconstruction and aids in treatment planning. It helps differentiate type A from type B dissection, may localize the intimal entry site and helps assess branch-vessel involvement and compromise and the relationship of the branch vessels to the true or false lumen. This information aids in planning treatment with either root replacement, intravascular stent placement, or fenestration [20].

Triple-rule-out CT is a new protocol used to assess the aorta, coronary arteries, pulmonary arteries, and the middle and lower portions of the lungs during a single scan with use of several optimally timed boluses of contrast material and ECG gating in patients who are at low risk for acute coronary syndrome. The aim is to minimize the contrast material dose and radiation exposure while achieving optimal image quality, providing coronary artery image quality equivalent to that of dedicated coronary CT angiography; pulmonary artery image quality equivalent to that of dedicated pulmonary CT angiography and high quality images of the thoracic aorta without pulsation artifact. In an appropriately selected emergency department patient population, triple-rule-out CT can safely eliminate the need for further diagnostic testing in over 75% of patients [21]. 

## 4. Conclusion

In conclusion, AD is a very emergent condition of the aorta and needs early diagnosis and prompt treatment for a successful outcome. CT imaging of the aorta is widely available and fast enough which provides an accurate diagnosis in unstable patients.
